# A New Approach for Predicting Strength Based on Temperature-Time History Using Two-Parameter Maturity ANN Models

**DOI:** 10.3390/ma17246157

**Published:** 2024-12-17

**Authors:** Jerzy Wawrzeńczyk

**Affiliations:** Faculty of Civil Engineering and Architecture, Kielce University of Technology, Al. Tysiąclecia Państwa Polskiego 7, 25-314 Kielce, Poland; zmsjw@tu.kielce.pl

**Keywords:** concrete, mortar, maturity, strength development, modelling, artificial neural networks

## Abstract

One widely used method to predict concrete strength development based on temperature variations during curing is the equivalent maturity time (te) method. This method uses the activation energy (Ea) as its key parameter, which reflects the cement’s sensitivity to temperature. However, research shows that the Ea value varies depending on factors such as cement type, water/cement ratio, temperature, and additives. The permanent subject of discussion is the question of what value of the Ea parameter should be assumed. In this paper, a new approach is proposed by using a neural network analysis method to develop a strength–temperature history model. It was assumed that the ANN-fc% = f(Q, E, T, t) model would have 4 inputs: hydration heat (Q), activation energy (Ea), temperature (T), and time (t). The research was conducted on mortars using 6 cements, at curing temperatures ranging from 5 to 35 °C, assessing strength over a 90 day period. The results showed that the ANN analysis method allows for estimating the relative compressive strength with sufficient accuracy. Analysis of the input nodes indicated that Q influences early strength gain, while Ea affects later strength development. The application of the ANN model for calculating strength based on temperature changes during maturation was illustrated.

## 1. Introduction

Compressive strength is the primary criterion for concrete quality control, as it effectively reflects the integrity of its internal structure and is a relatively straightforward technical test. Since World War II, there has been significant interest in methods for rapidly assessing in situ concrete strength by monitoring temperature changes over time. The theoretical foundations and experimental results of these methods have been extensively documented in various studies, including review publications [1,2]. The literature on concrete maturity contains numerous studies on predicting early-age strength [3,4], as well as many investigations into predicting late-age strength using modified maturity models [5]. Several models have been developed addressing two key issues: the maturity index and temperature–time relationship and the strength–maturity relationship (Table 1).

In general, when modelling the strength–maturity relationship in concrete, some researchers have prioritised creating simplified mathematical descriptions, while others have focused on minimising estimation errors through regression analysis of test data [1,6]. Additionally, many authors [4,5,7] have proposed the use of correction factors to improve the accuracy of these models.

The Hansen and Petersen function [8] is regarded as one of the most advanced models, linking concrete strength to the maturity measure, represented as the equivalent maturing time (te). This function, similar to the Arrhenius function, includes a parameter called activation energy (Ea), which reflects a given cement’s sensitivity to curing temperature. While this concept is straightforward, it is unlikely that a single parameter can fully capture the complexities of a material as intricate as cement.

**Table 1:** Selected models used in the theory of maturity-strength of concrete.

Maturity Estimation Model	
Nurse-Saul [9]	*M* = ∑(*T* − *To*)·Δ*t*,
Rastrup [10]	*te* = ∑(*T* − *To*)·(*Tr* − *To*)·Δ*t*
Hansen and Pedersen [8]	*te* = ∑exp(−*E*/*R*)[1/*T* − 1/*Tr*]·Δ*t*
Carino et al. [11]	*te* = ∑exp{*B*(*T* − *Tr*)}·Δ*t*
Strength Prediction Model	
Plowman [12]	*S* = *a* + *b*·log(*M*)
Lew and Reichard [13]	*S* = *K*/{1+*K*·*a*·[log(*M* − 30)] *b*}
Hansen and Pedersen [14,15]	*S* = *Su*·exp{−[*τ*/*M*]*α*}
Gompertz [16]	*S* = *Su*·exp(−*a*·*e* − *b*·log*M*)
ASTM C 1074 [17,18]	*S* = *Su·k*·(*t* − *to*)/{1 + k·(*t* − *to*)}

Various analyses of theoretical and experimental results often yield differing conclusions regarding the value of the Ea coefficient. This value depends not only on the type of cement but also on the water-to-cement ratio (e.g., OPC vs. HPC concrete), temperature [19], and the presence of additives and admixtures.

The Ea coefficient can be determined through strength tests [18,20] or by calorimetric measurements of the heat released during hydration [21]. Wirtquin [21,22] proposed a method for determining activation energy by measuring cement hydration heat. Earlier, Venuant [23] demonstrated a strong correlation between hydration heat and mortar strength. Similarly, Kurdowski and Pichniarczyk [24] found a strong correlation between the hydration heat of various CEM I cements and their strength after 2 and 28 days.

For more complex relationships, an alternative to traditional statistical methods (where the functional form is known) is the use of artificial neural networks (ANNs). These “black box” methods have been successfully applied for over 30 years to solve various material and technological challenges [25]. ANN analysis has been used to model the relationship between mortar strength and various compositional factors [26,27,28,29,30].

In this paper, a different approach is explored to address strength-maturity relationships. An ANN model was developed as an implicit form of the regression function to encompass the entire problem. The research aimed to answer the following questions:

(a) Can the ANN method produce a model that accurately describes the relationship between mortar strength, temperature, and time?
(b) Does incorporating two predictive parameters—heat of hydration (Q) and activation energy (Ea)—into the model improve its description of mortar strength development, both at early and late ages?
(c) How can the ANN-based model, fc% = f(Q, E, T, t), be used to calculate the relative strength of mortar as it depends on variations in curing temperature and time?

## 2. Materials and Methods

The research programme (Table 2) involved measuring the compressive strength of the mortar cured at three different temperatures: 5 °C, 20 °C, and 35 °C. Six series of samples (mortar mixes) were prepared using six different cements (Table 2). Based on the strength tests, the activation energy (Ea) for each cement was determined, and in the calorimetric tests, the amount of heat of hydration (Q) after 48 h of hydration was measured. The data collected formed a dataset used to develop a regression model through artificial neural network (ANN) analysis. The development of the ANN-fc% = f(Q, E, T, t) model aimed to predict the strength development by considering two cement parameters (Q and E) in relation to the temperature history (T) over time (t). The proposed approach sought to create a model that covers a broader range of predictive parameters (Q, E) that characterise different cements. This single model would address both the maturity index and the strength–maturity relationships.

### 2.1. Materials

The mortars were prepared using the following materials:

- Standard sand (0–2 mm grain size) in accordance with CEN PN-EN 196-1.
- Six different cements: five Portland cements (CEM I) and one slag cement (CEM III/A).
- A plasticiser.

The characteristics of the cements used are presented in Table 3.

In addition, calorimetric tests were conducted on the six cements using an isothermal calorimeter to determine the amount of heat released during hydration at 20 °C. Table 3 shows the recorded heat (Q) after 48 h of testing.

### 2.2. Mixture Proportions and Sample Preparation

Mortar tests were carried out according to the procedure described in ASTM C1074-04 [29]. The reference material was structural concrete with a water-to-cement ratio (W/C) of 0.45 and a cement content (C) of 378 kg. The proportions of the mortar mixture were kept constant, using the same fine aggregate-to-cement ratio, W/C ratio, and plasticiser-to-cement amounts as in the concrete mix. The mixing and preparation of the samples were carried out at room temperature (approximately 22 °C).

Six series of samples were prepared using different cements, with the series labelled according to the cement used, designated by letters A to F. In each case, a batch of mortar, approximately 15 dm^3^ in volume, was mixed and formed into 15 moulds, each containing three beams measuring 40 × 40 × 160 mm, totalling 45 samples. The samples were divided into three groups of five moulds, which were immediately placed in water baths. The samples were first placed in water baths with water temperatures of 5 °C and 35 °C, followed by 20 °C. The metal moulds were partially submerged in water, about halfway up their height, to ensure the rapid temperature stabilisation of the mortar. After 1–2 days, the samples were demoulded and placed back into the water, where they were stored until compressive strength testing. The water in the bath was continuously circulated and kept at the specified temperature.

### 2.3. Compressive Strength

The compressive strength tests for the hardened mortar were performed according to PN-EN 196-1 standards. For each mortar type, strength was measured after 1, 3, 7, 14, 28, 56, and 90 days of curing at 5 °C, 20 °C, and 35 °C. The samples were stored in water baths at the corresponding temperatures until compressive tests were performed. For further analysis, the compressive strength was taken as the average of four measurements.

**Figure 1:**
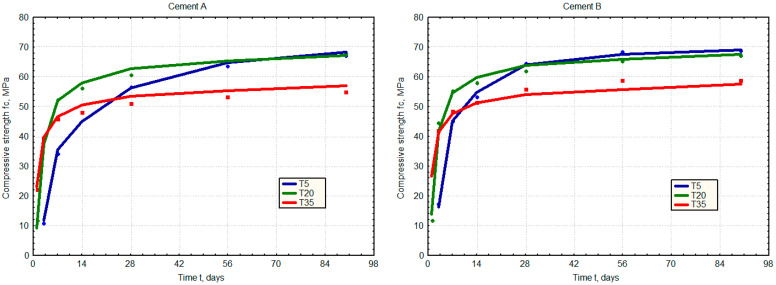
Relationships between the compressive strength of mortars (Series A–F), the cure temperature (5 °C, 20 °C and 35 °C), and time. The results of the compressive strength tests are marked as points, while the solid lines correspond to the values approximated by the ANN-fc model.

The results were plotted as points in Figure 1. In the next stage of the calculations, the relative strength of the mortar was analysed as the ratio of the measured strength at a given time and the strength achieved by that mortar after 90 days of curing at 20 °C.

On the basis of the strength tests, the activation energy (Ea) of the six cements was determined using the procedure described in ASTM C1074. For further analysis, the parameter referred to as the “activation temperature” (E), which is the activation energy divided by the gas constant, was used. The calculation results are presented in Table 4.

## 3. Results and Discussion

### 3.1. ANN Model for Prediction of Strength Development

The complex nature of the relationships between the factors under investigation makes it challenging to develop an analytical model that accurately describes their effects on the strength of the material. Therefore, an attempt was made to use artificial neural network (ANN) analysis for this purpose, utilising the QNET’97 software version 1.0 [31]. In this approach, the ANN method serves as an alternative to multidimensional regression analysis. The developed neural network represents an implicit non-linear function (a “black box” relationship).

Backpropagation neural networks require that all training targets be normalised between 0 and 1, as the output node signals are restricted to this range. QNET also requires input normalisation to enhance training performance. The software automatically performs data normalisation, ensuring that all input data and training targets are scaled between 0.15 and 0.85. The network outputs are then automatically adjusted to the appropriate range.

The analysis focused on the influence of four input factors on the development of compressive strength in hardened mortar (fc%):

- Q: Hydration heat of the cement after 48 h, J/g,
- E: Activation temperature of the cement, °C,
- T: Temperature of the mortar, °C,
- t: Curing time, days.

The information on the range of variability for each of these factors is presented in Table 5.

**Figure 2:**
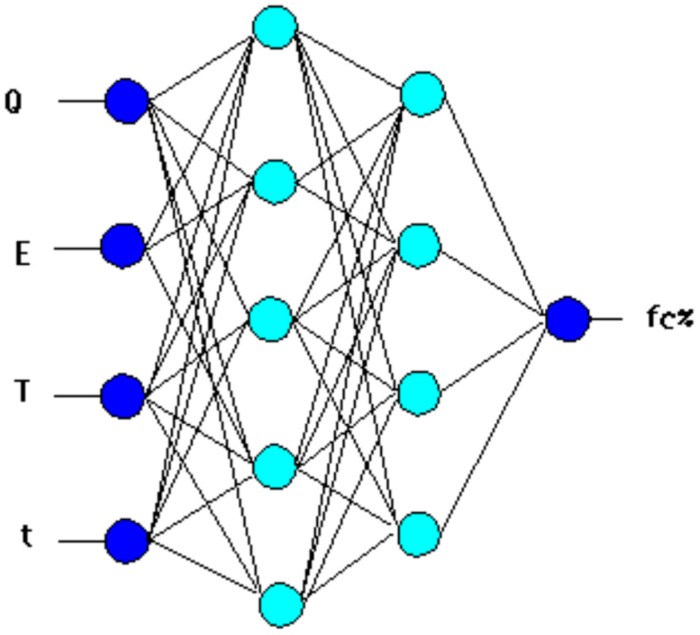
Diagram of the four-layer structure of the ANN-fc% neural network.

After conducting a series of trials, a network with a 4-[5-4]-1 structure was developed (Figure 2).

Initially, the parameters for the neural network were determined, and the calculated compressive strength values (fc) are presented as solid lines in Figure 1. Then, following the suggestion of Tang and Carino, the relative strength values of the mortar were calculated by dividing the compressive strength by the corresponding strength at 90 days of curing at a temperature of T = 20 °C (fc% = fci/fc90).

**Figure 3:**
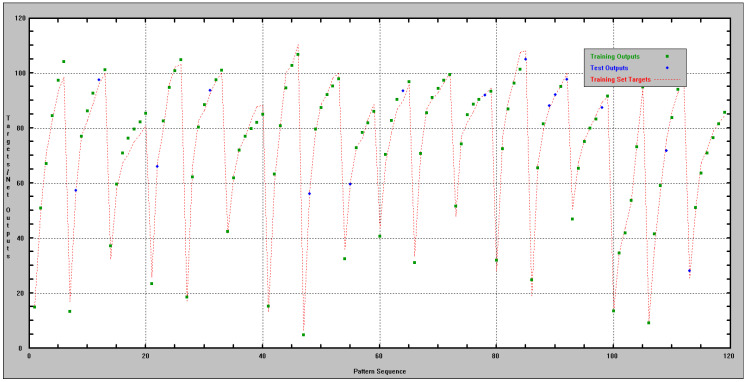
Comparison of target/net outputs versus pattern sequence.

**Figure 4:**
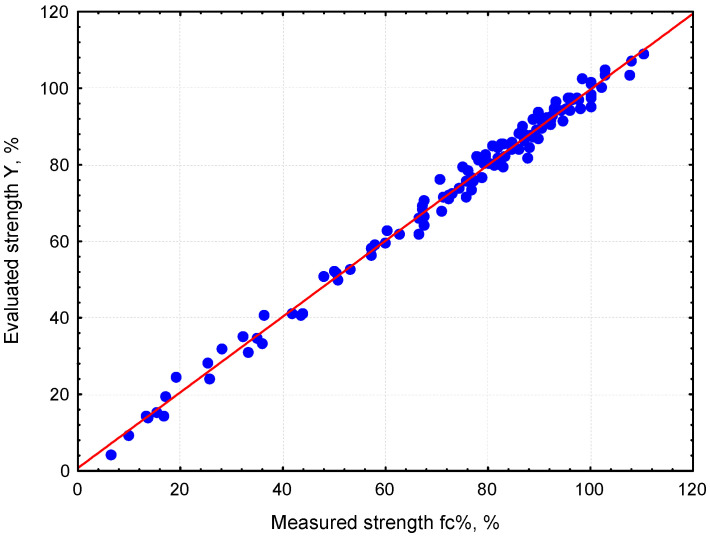
Measured strength fc% versus ANN-fc% outputs.

A comparison of the target and network outputs versus the pattern sequence is shown in Figure 3. The comparison between the measured strengths and the fc% values determined using the ANN-fc% network is illustrated in Figure 4.

The precision of the estimation related to the training and testing of the dataset is presented in Table 6.

Analysing the influence of input data on the output value of fc%, it can be concluded that the most significant factors are the curing time (t) and temperature (T), followed by the heat of hydration (Q), and, finally, the activation parameter (E).

**Figure 5:**
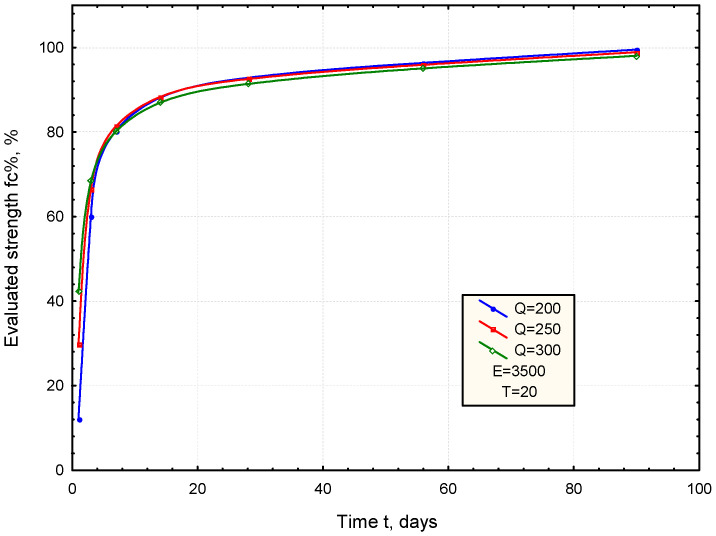
The effect of cement parameter (Q) on the development of the mortar strength (E = 3500 °C, T = 20 °C).

**Figure 6:**
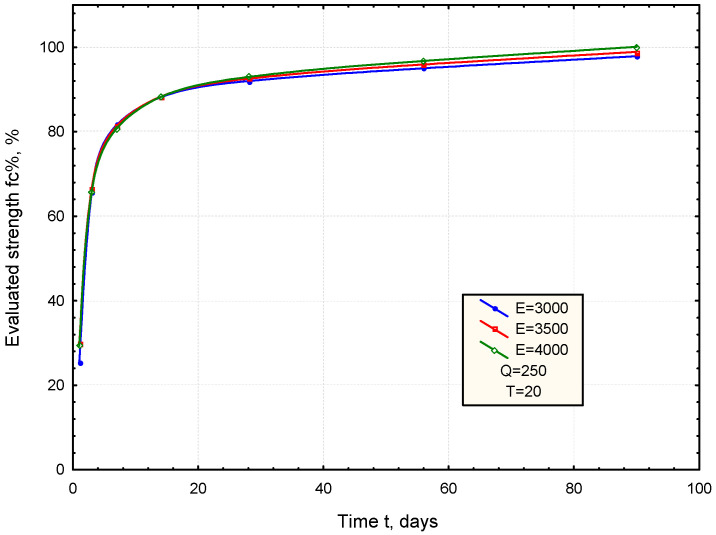
The effect of cement parameter (E) on the development of the mortar strength (Q = 250 J/g, T = 20 °C).

An analysis of the model was performed to evaluate the effect of cement parameters (Q, E) on the development of the mortar strength at constant temperature (T = 20 °C). Varying the value of Q (hydration heat) within the range of 200–300 J/g, while keeping the E constant at 3500 °C, shows a significantly higher increase in early strength (Figure 5). On the other hand, varying the parameter E from 3000 to 4000 °C, assuming a Q value of 250 J/g, reveals a notable influence of the E parameter during the later stages of mortar curing (Figure 6).

From the analysis of the relationships obtained, it can be inferred that the Q parameter (hydration heat) has a greater influence on early strength gain during the initial curing period, while the E parameter (activation temperature) impacts the development of the strength in the later stages of curing.

This paper provides an example of the direct application of the developed model to calculate the strength of a given mortar, depending on the temperature and the curing time. The approach presented in this study can be applied to predict concrete strength in both early and later stages of curing. The results demonstrate that the ANN analysis method is an effective technique for modelling such complex problems and allows for estimating relative compressive strength (fc%) with sufficient accuracy.

### 3.2. Application of the Developed ANN-fc% Network for Predicting Hardened Mortar Strength

**Figure 7:**
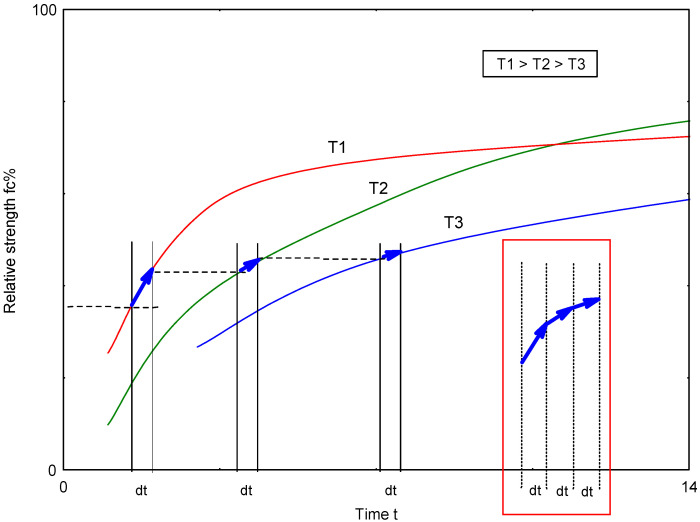
Schematic showing how fc% strength is calculated as the sum of strength increments in successive time steps, accounting for changes in curing temperature.

The determination of ANN-fc% parameters enables the prediction of the relative strength of mortar depending on temperature and curing time. The method to determine fc% is performed iteratively, as illustrated in the conceptual diagram (Figure 7):

- Calculations are performed for a fixed time step dt.
- Within the interval dt at a given temperature, the strength increment is dYi (%).
- After time t = n × dt, the strength level is Y at the current temperature T1.
- If the temperature changes to T2 in the next time step, the time at which the strength level Y is reached at this temperature must be calculated, along with the strength increment dY2.
- In subsequent time steps, further strength increments dYi are calculated.
- The increase in strength with changing temperature cannot be negative dY ≥ 0.
- The final strength Y after a certain time t is the sum of the individual increments dYi, as shown in the lower right corner of the diagram.
- The process is repeated until the final time is reached.

A simple calculation programme was prepared as a macro in Statistica 13.0 [32] (visual basic STB) to perform these calculations. Initially, input data characterised the cement, such as the heat of hydration (Q) and the activation parameter (E), and the mortar temperature history (T) during curing (t). On the basis of these data, the QNET programme is called to calculate the fc% value, which is then transferred to the Statistica spreadsheet.

**Figure 8:**
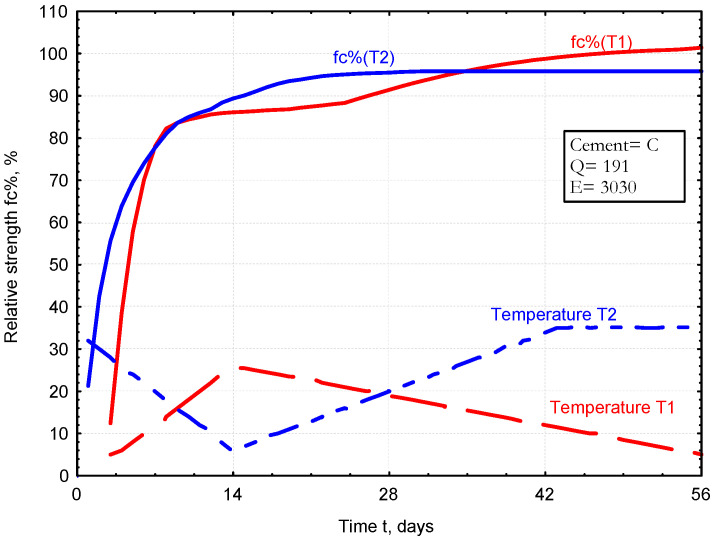
Development of relative strength (fc%) during curing at variable temperatures.

The data collected in the spreadsheet are used to create a graph showing the development of the relative strength of the mortar (fc%) during curing at varying temperatures. Examples illustrating the change in the relative strength of the mortar (made of cement C) for two different temperature curves, T1 and T2, are shown in Figure 8.

In this study, a method for calculating fc% is proposed using two programmes: Statistica and QNET. Statistica macro is the basic programme used for calculations and graphical presentation of results using (GUI), while QNET is used to calculate the fc% value.

The appendix attached as “Supplementary Materials” shows how to perform calculations without using QNET, describing in detail the matrix calculations and transformations of weight values derived from the sigmoid transfer function between the network layers. Knowing the weight matrices for a given model, one can perform calculations without QNET (QNET was designed for 32-bit operating systems and does not run on newer computers). A script created according to this scheme can be integrated as a subroutine/function in the main calculation programme, e.g., Excel or another programme.

## 4. Conclusions

The aim of the research was to develop a new type of model: fc% = f(Q, E, T, t), which allows for estimating the relative strength of mortar, simultaneously taking into account two parameters characterising the given cement: activation energy (Ea) and measured heat of hydration (Q). To achieve this, the artificial neural network (ANN) method was applied as a technique for multifactor regression analysis. As a result of the analysis, an ANN model was developed, representing an implicit non-linear “black box” function. The results showed that the ANN analysis method is an efficient technique for modelling such complex problems.

The developed ANN-fc% model has 4 inputs, including heat of hydration (Q), activation energy (Ea), temperature (T), and time (t). In order to collect training data, an extensive mortar testing programme was conducted using six cements, curing temperatures from 5 to 35 °C, and 90 day strength. The results showed that the ANN analysis method allows for estimating the relative compressive strength with sufficient accuracy. Input nodes analysis showed that hydration heat (Q) influences early strength gain, while activation energy Ea affects later strength development. The method of using the ANN-fc% model to calculate the relative strength of mortar depending on the temperature change during maturation was shown. The approach presented in this paper could be useful for quality control and non-destructive prediction of concrete strength.

When treating the presented research as a preliminary phase, it is necessary to conduct further studies to verify the relationships obtained based on the testing of concrete samples.

## Figures and Tables

**Table 2:** The experimental variables.

Experimental Level	Items
W/C ratio	0.45
Cement	CEM I, CEM III Heat of hydration Q
Curing temperature, °C	5, 20, 35
Compressive strength, MPa	1, 2, 3, 7, 14, 28, 56, 90 days Activation energy Ea->E

**Table 3:** The characteristics of the cements.

Cement		A	B	C	D	E	F
Class		CEM I 32.5R	CEM I 42.5R	CEM I 42.5R	CEM I 52.5R	CEM I 42.5R	CEM III/A 42.5
CaO	%	62.4	63.3	62.8	63.5	63.2	52.5
SiO_2_	%	21.3	19.8	19.0	19.4	21.9	27.7
Al_2_O_3_	%	5.0	4.9	5.4	5.4	3.7	6.0
Fe_2_O_3_	%	3.1	2.6	2.9	3.0	4.8	1.7
SO_3_	%	2.7	2.6	3.2	3.3	2.5	2.7
Na_2_O eq	%	0.47	0.71	0.8	0.8	0.66	0.79
Density	kg/dm^3^	3.05	3.10	3.11	3.12	3.14	2.96
Blain suf.	m^2^/kg	290	362	329	509	373	411
Strength fc-2	MPa	15,2	29.2	26.1	41.1	29.9	21.5
fc-28	MPa	47,3	56.2	56.7	65.4	55.1	53.2

**Table 4:** Activation temperature E and heat of hydration Q of tested cements.

		Cement					
		A	B	C	D	E	F
Activation temperature E	°C	4450	4300	3030	2760	3100	4800
Heat of hydration Q	J/g	196	214	191	302	244	173

**Table 5:** Provides information on the range of variability for each of these factors.

Input/Output	Unit		Min	Max
Q	J/g	X1	173	302
E	°C	X2	2760	4800
T	°C	X3	5	35
t	days	X4	1	56
fc	MPa	Y1	3.8	74.3
fc%	%	Y2	6.6	110.3

**Table 6:** Estimation of ANN-fc% model errors.

	N	Max. Error	RMS Error	Correlation
Training set	104	6.03	0.0181	0.994
Test set	15	5.30	0. 0137	0.956

## Data Availability

The original contributions presented in the study are included in the article/Supplementary Material, further inquiries can be directed to the corresponding author/s.

## Supplementary Materials

The following supporting information can be downloaded at: https://www.mdpi.com/article/10.3390/ma17246157/s1, the appendix attached as Supplementary Materials shows how to perform calculations without using the ANN programme detailing the matrix calculations and transformations derived from the sigmoid transfer functions between network layers.
