# Supplementary material for: A New Approach for Predicting Strength Based on Temperature-Time History Using Two-Parameter Maturity ANN Models
Source: Materials (Basel). 2024 Dec 17;17(24):6157. doi: 10.3390/ma17246157 (PMC11678383; doi:10.3390/ma17246157)
Supplement: Supplementary file 1 [file materials-17-06157-s001.zip › materials-3290938-supplementary.pdf]

Załącznik 1 – Obliczenia według sieci ANN-fc%

Sposób normalizowania danych wejściowych i wyjściowych

		XMin	XMax
X1	Q [J/g]	173	302
X2	E [kJ/mol]	2760	4800
X3	T [°C]	5	35
X4	t [days]	1	56
Y1	fc [MPa]	3.8	74.3
Y2	fc% [%]	6.6	110.3

X(i)=0.15+0.7*$\frac{(x-Xmin)}{(Xmax-Xmin)}$

MATRIX_A = $\begin{bmatrix} -13.659 & 3.00066 & -3.69268 & -4.46159 & 1.7884 \\ 0.53041 & -0.12695 & 2.20964 & 21.5926 & -4.47981 \\ -2.57126 & -4.3655 & 17.3973 & 3.24461 & -3.26951 \\ -2.92825 & 17.0543 & -5.78064 & -3.46922 & 3.82515 \\ -0.18638 & 0.60554 & -4.97054 & 1.32218 & 1.4326 \end{bmatrix}$

MATRIX_B = $\begin{bmatrix} -2.51689 & 1.9439 & 1.99901 & -1.04831 & 2.85668 & -2.73523 \\ -4.87607 & -2.19247 & -0.996297 & -1.2223 & -5.39183 & -1.69398 \\ -8.17001 & 1.22619 & -1.02763 & 5.60401 & 3.23156 & -9.46689 \\ -1.25124 & -19.436 & -1.87757 & -1.55248 & -10.0811 & 14.7646 \end{bmatrix}$

MATRIX_C = $[2.29488 \quad -3.86384 \quad 1.28727 \quad -10.9542 \quad -0.874625]$

X = $\begin{bmatrix} x1 \\ x2 \\ x3 \\ x4 \\ 1 \end{bmatrix}$ [A]*[X]= [W1]   W1 = $\begin{bmatrix} w1(1) \\ w1(2) \\ w1(2) \\ w1(4) \\ w1(5) \end{bmatrix}$ w1sign(i)=$\frac{1}{1+Exp(-w1(i))}$

W1sign = $\begin{bmatrix} w1sign(1) \\ w1sign(2) \\ w1sign(2) \\ w1sign(4) \\ w1sign(5) \\ 1 \end{bmatrix}$ [B]*[W1sign]=W2   W2 = $\begin{bmatrix} w2(1) \\ w2(2) \\ w2(2) \\ w2(4) \end{bmatrix}$

w2sign(i)=$\frac{1}{1+Exp(-w2(i)}$ W2sign= $\begin{bmatrix} w2sign(1) \\ w2sign(2) \\ w2sign(2) \\ w2sign(4) \\ w2sign(5) \\ 1 \end{bmatrix}$

[C]*[W2sign]=Y

fc% = $\frac{(Y-0.15)}{0.7}$* $(Ymax - Ymin)$
